# Regulatory Mechanisms of Injury and Repair after Hepatic Ischemia/Reperfusion

**DOI:** 10.6064/2012/513192

**Published:** 2012-09-20

**Authors:** Alex B. Lentsch

**Affiliations:** Department of Surgery, College of Medicine, University of Cincinnati, 231 Albert Sabin Way, ML 0558, Cincinnati, OH 45267-0558, USA

## Abstract

Hepatic ischemia/reperfusion injury is an important complication of liver surgery and transplantation. The mechanisms of this injury as well as the subsequent reparative and regenerative processes have been the subject of thorough study. In this paper, we discuss the complex and coordinated responses leading to parenchymal damage after liver ischemia/reperfusion as well as the manner in which the liver clears damaged cells and regenerates functional mass.

## 1. Introduction

Hepatic ischemia/reperfusion (I/R) injury is a consequence of vascular inflow occlusion due to portal vascular clamping during complex liver surgery. I/R injury of the liver is directly related to the duration of liver ischemia and is a major cause of morbidity and mortality from liver transplantation and resection [1–3]. There has been considerable study of the biochemical and cellular changes occurring during I/R which has informed clinical practice. The results of these studies, which are the focus of this paper, have led to advances in our understanding of the pathophysiology of hepatic I/R injury and the development of new therapeutic modalities. 

## 2. Initiation of Reperfusion Injury

Early work by Jaeschke et al. [4–6] established that there are two distinct phases of liver injury after warm I/R. The initial phase of injury occurring within the first couple of hours of reperfusion is characterized by Kupffer cell-induced oxidant stress. Kupffer cell production and release of reactive oxygen species, including superoxide anion and hydrogen peroxide, result in acute hepatocellular injury. Blockade of Kupffer cell activity, accomplished by administration of gadolinium chloride or methyl palmitate, reduces acute hepatocyte damage. In addition, complement activation products are critically important for Kupffer cell activation during the initial phase of injury as depletion of complement reduces Kupffer cell-induced oxidant stress [7]. Despite the contribution of Kupffer cell-derived oxidants, the extent of injury during this initial phase is far less than that observed at later time points. Events occurring during the initial phase of liver injury, including activation of Kupffer cells, initiate a complex inflammatory cascade leading to the recruitment of various populations of leukocytes to the liver. The first population of leukocytes recruited after reperfusion is CD4 T lymphocytes.

## 3. Hepatic Recruitment of CD4 T Cells

Significant involvement of T lymphocytes in hepatic I/R was first demonstrated in 1997 in a report that found that T lymphocytes rapidly accumulated in the liver after reperfusion [8]. This study showed that CD4, but not CD8, T lymphocytes were recruited into the postischemic liver within 1 hour of reperfusion. The briskness of this response is surprising as it preceded the influx of innate immune cells to the injured tissue. Later studies by our group confirmed this rapid recruitment of CD4 T cells [9]. The mechanisms by which T cells are so rapidly recruited to the postischemic liver remain undefined. However, there is growing evidence that hepatic expression of chemokines is an important contributor to this process [10]. 

As mentioned above, CD4 lymphocytes are recruited into postischemic liver long before any appreciable neutrophil accumulation. Both antibody depletion of CD4 T cells and CD4-knockout mice showed reduced liver recruitment of neutrophils after I/R [8, 9]. The mechanism by which CD4 T cells regulate subsequent neutrophil accumulation appears to be related to their release of IL-17. IL-17 is preferentially expressed and secreted by activated CD4 lymphocytes [11]. Furthermore, in a model of peritoneal inflammation, IL-17 was found to mediate neutrophil recruitment by increasing the production of chemokines by the peritoneal mesothelium [12]. IL-17 has also been shown to induce chemokine production by other cell types, including epithelial cells, fibroblasts, osteoblasts, and endothelial cells [13–15]. Our studies found that production of neutrophil-attracting chemokines was decreased in CD4-knockout mice and that in wild-type mice treated with anti-IL-17 antibodies, chemokine expression was reduced [9]. In both of these experiments, liver neutrophil accumulation was also reduced. Moreover, adoptive transfer of CD4 lymphocytes into CD4-knockout mice resulted in dramatic increases in the expression of chemokines and the degree of liver neutrophil recruitment [9]. Thus, it would appear that CD4 lymphocytes are an important regulator of hepatic neutrophil recruitment during liver I/R and that this occurs via their release of IL-17.

The question of whether or not T cell involvement in liver I/R is driven by antigenic or nonantigenic mechanisms has not been elucidated. Some studies show that utilization of MHC II blocking antibodies has no effect on serum ALT following hepatic IR [16]. This study suggested that T cells play a beneficial role not involving the *αβ* TCR and that lymphocyte actions occur through a nonantigenic mechanism. It is well established that during hepatic I/R inflammatory cytokines such as IL-12 and IL-18 are rapidly expressed [17, 18]. Furthermore, nonnaive as well as unconventional T cells can be functionally activated by these cytokines in a manner independent of TCR engagement [19–21]. Taken collectively, these studies suggest the possibility of nonantigenic activation of T cells during the initial stages of I/R in the liver. Alternatively, recent studies in other models of I/R, have discovered the presence of an IgM that reacts with self-antigens generated by damaged tissues [22, 23]. These self-reactive IgMs activate the classical pathway of complement and contribute substantially to the initiation of the injury response. A similar mechanism may be applicable to liver I/R, but to date has not been examined.

In order to successfully mount an immune response to an antigen, T lymphocytes need to receive two different signals. The first signal is delivered by the antigen upon its binding to the T-cell receptor (TCR). This antigen-specific event is usually termed signal one. The second signal, signal two, is costimulation delivered by antigen presenting cells and is a non-antigen-specific event. There are a large number of different costimulatory molecules and they vary greatly in their expression patterns and function [24]. One of the most widely studied co-stimulatory pathways is the CD40-CD154 pathway. CD40 is a member of the tumor necrosis factor receptor superfamily and is expressed on APCs such as dendritic cells (DCs), macrophages, and B-cells. Ligation of CD40 by its cognate ligand CD154 (which is transiently expressed on activated T helper cells) leads to co-stimulation of the target cell. Specifically, during liver I/R, it has been shown that gene therapy-mediated CD154 blockade (Ad-CD40 Ig), antibody-induced systemic CD154 blockade (MR1 mAb), and genetically targeted CD154 absence (CD154 KO mice) ameliorated otherwise fulminant injury in a warm liver I/R model [25]. These beneficial effects resulting from the disruption of CD154-CD40 signaling were accompanied by (1) diminished liver T-cell sequestration; (2) decrease of VEGF expression (3) inhibition of TNF-*α* and T-helper (Th) type 1 cytokine production and (4) induction of antiapoptotic (Bcl-2/Bcl-xl) and depression of proapoptotic (caspase-3) proteins. 

Another widely studied co-stimulatory pathway is the CD28/CD80/86 pathway. CD28 is constitutively expressed on T cells. The ligands for CD28 are CD80 and CD86 (B7-1, B7-2), both members of the immunoglobulin (Ig) superfamily, which are transiently expressed on activated APCs. Both CD80 and CD86 are increased in the liver after I/R [26, 27]. Ligation of CD28 by these molecules in conjunction with antigen recognition via the TCR complex leads to activation of the T cell. An additional feature of this pathway is the existence of an alternative receptor for CD80/86 called CD152 (CTLA-4), which unlike CD28, is upregulated after T cell activation and results in suppressive T cell function. Indirect evidence for a critical role for T cells in kidney I/R came from blocking one of the costimulatory pathways necessary for T-cell activation. Blocking the B7-CD28 costimulation pathway by CTLA4 Ig, a recombinant fusion protein, containing the extracellular domain of human CTLA4 (a homologue of CD28), resulting in T cell anergy, ameliorated renal dysfunction and decreased mononuclear cell infiltration in a model of renal cold ischemia [28]. It has yet to be elucidated whether such treatment during liver ischemia reperfusion would yield similar results.

The liver sinusoidal endothelial cell (LSEC) has been described as a new type of APC that resides in the liver [29, 30]. LSEC is also believed to express the costimulatory moieties CD40, CD80, and CD86 and stimulate T cells through peptide presentation in the context of MHC class I and II molecules [22, 31]. This would allow endothelial activation by T cells and vice versa, due to TCR-MHC and either CD40-CD154- or CD28-B7-dependent pathways. However, in a contrary report that compared LSEC and dendritic cells directly, it was found that LSEC expressed surface markers only reflective of an endothelial phenotype. Further, highly purified LSEC had undetectable levels of the co-stimulatory receptors CD40, CD80, and CD86 and only minimal MHC class II. This paper concluded that LSECs are poor stimulators of T cells, but other properties, such as their high capacity for antigen uptake and direct access to circulating lymphocytes, may enable them to contribute to the unique immunologic function of the liver [32].

The precise manner in which intrahepatic T cells interact with various APCs in the liver during the response to I/R has not been elucidated. However, the rapid recruitment of lymphocytes to the liver coincides with the induction of a robust hepatic inflammatory response.

## 4. Initiation and Propagation of Inflammation in the Liver by I/R

The hepatic inflammatory response to I/R appears to begin with the elaboration of the cytokines, IL-12 and IL-23. Increased expression of these cytokines, at both the mRNA and protein levels, can be detected prior to hepatic reperfusion and this expression is short-lived, disappearing within 4-5 hours of hepatic reperfusion [18, 33]. The importance of IL-12/23 in the initiation of the hepatic inflammatory response to I/R was demonstrated in studies using both neutralizing antibodies as well as mice lacking the p40 subunit, which is common to both IL-12 and IL-23. In both experiments, a functional lack of IL-12 prevented increased expression of tumor necrosis factor-alpha (TNF*α*) and subsequent development of neutrophil-dependent liver injury. This prominent role of IL-12/23 was direct and not mediated by induction of interferon-gamma (IFN*γ*) production, as mice nullizygous for IFN*γ* were indistinguishable from wild-type mice in their response to hepatic I/R [18].

The liver cell population responsible for producing IL-12/23 has not yet been identified, but Kupffer cells and stellate cells are likely sources [34]. Similarly, the signalling mechanism through which IL-12/23 acts to illicit TNF*α* production remains undefined. IL-12/23 is known to be a potent activator of the transcription factor, signal transducer and activator of transcription-4 (STAT4) [35, 36]. However, STAT4-deficient mice were not protected from liver I/R injury [37], suggesting that another signalling mechanism exists that is responsible for the in vivo gene induction of TNF*α* by IL-12/23. 

The production of TNF*α* and IL-1 by Kupffer cells after I/R has long been thought to be the primary initiating events for propagation of the hepatic inflammatory response [38–41]. We now know that expression of IL-12/23 is required for the full expression of TNF*α* and that IL-1 plays only an accessory role in the hepatic inflammatory response to I/R. As with many other acute inflammatory responses, TNF*α* is a central mediator in the hepatic response to I/R. Liver production of TNF*α* does not occur during hepatic ischemia but begins to increase shortly after reperfusion at a time when hepatic IL-12/23 levels are maximal [18]. The importance of TNF*α* in the hepatic inflammatory response has been well described and blockade of this mediator abolishes liver inflammation and hepatocellular injury [38, 42]. These protective effects were subsequently found to be attributed to TNF*α* induction of secondary inflammatory mediators. Blockade of TNF*α* prevents the expression of hepatic vascular adhesion molecules as well as the expression of CXC chemokines, which are chemotactic for neutrophils [43–46].

IL-1 was presumed to share many of the same effects of TNF*α*, based on an early study showing that prophylactic treatment with IL-1 receptor antagonist protected against hepatic I/R injury [41]. However, a more recent study indicates that IL-1 functions to augment neutrophil recruitment but does not play an essential role in the development of liver inflammation after I/R [47]. In this study, IL-1 receptor-knockout mice experienced the same degree of hepatocellular injury as their wild-type counterparts, but had reduced neutrophil accumulation in the liver. This was found to be associated with attenuated activation of the transcription factor, NF-*κ*B, and reduced expression of CXC chemokines. It appears that IL-1 functions to augment the inflammatory response at later time points through the induction of CXC chemokine expression, but the lack of IL-1 function does not significantly affect the development of liver inflammation.

## 5. Transcriptional Activation of Proinflammatory Cytokines

A common theme amongst each of the three “early response” cytokines discussed above is their gene regulation. The gene expression of IL-12/23, TNF*α*, and IL-1 is controlled, at least in part, by the transcription factor, NF-*κ*B [48]. 

 Other transcription factors, such as AP-1 and members of the signal transducer and activator of transcription (STAT) family, also regulate proinflammatory mediator expression. However, their roles during hepatic I/R injury are less well studied. The term NF-*κ*B refers to proteins of the Rel family which share a homologous amino acid sequence in their amino termini called the Rel homology domain that is necessary for dimerization, DNA binding and I*κ*B (inhibitor of NF-*κ*B) binding, [49, 50]. These proteins bind to form homo- or heterodimers with different degrees of transcriptional activity [51]. The classical form of NF-*κ*B, and the dimer found most commonly in the liver, is a heterodimer composed of p50 and p65 [49]. In unstimulated cells, NF-*κ*B is sequestered in the cytoplasm by inhibitors of *κ*B (I*κ*B) proteins, of which there are currently at least 5 known isoforms. I*κ*Bs prevent nuclear localization of NF-*κ*B by masking its nuclear localization signal peptide and block NF-*κ*B from binding to DNA by allosteric inhibition [52].

Two modes of NF-*κ*B activation have been described to occur in the liver during I/R injury. The first mode is the classical pathway of NF-*κ*B activation in which cell stimulation results in the serine phosphorylation of I*κ*B by the I*κ*B kinase complex (IKK complex). This kinase complex consists of two catalytically active subunits, IKK*α* and IKK*β*, and a nonenzymatic regulatory scaffold protein I*κ*K*γ* (also known as NF-*κ*B essential modifier, NEMO) [53, 54]. Phosphorylated I*κ*B then becomes the target of ubiquitin ligase which polyubiquitinates the protein for subsequent proteasomal degradation [55, 56]. In addition to this well-characterized pathway, there appears to be an alternative method of NF-*κ*B activation that does not involve the IKK complex, serine phosphorylation, and proteosome mediated degradation of I*κ*B. This alternate mechanism of NF-*κ*B activation was originally described in hypoxic T cells and involves the phosphorylation of I*κ*B*α* on tyrosine residue 42 that leads to its dissociation from NF-*κ*B [57]. However, tyrosine-phosphorylated I*κ*B*α* is not proteolytically degraded. Experimental data suggest that activation of NF-*κ*B via this mechanism occurs predominantly after hypoxia, whereas the classical pathway occurs primarily after cytokine stimulation [58–61]. For both mechanisms of activation, once NF-*κ*B is freed from I*κ*B it translocates to the nucleus where it initiates the transcription of target genes. 

The activity of free NF-*κ*B is also tightly regulated. First, NF-*κ*B activates the transcription of its own inhibitor I*κ*B*α*, leading to the termination of NF-*κ*B response which is rapid but transient [56, 62]. Second, posttranslational modifications of NF-*κ*B subunits by phosphorylation and acetylation affect the transcriptional activity, stability, DNA binding affinity, and subcellular localization of NF-*κ*B [63–65]. In particular, the robust induction of NF-*κ*B requires the phosphorylation of p65 on its serine residue 276 which permits the subsequent recruitment of coactivator CBP/p300 [66–68]. Multiple other p65 phosphorylation sites involving multiple kinases have also been identified [63]. Overall, the phosphorylation of p65 appears to be essential for the optimal activity of NF-*κ*B. Similar to phosphorylation, acetylation of p65 occurs at multiple sites and affects the function of NF-*κ*B. For example, acetylation of lysine 221 on p65 enhances DNA binding and hinders association of I*κ*B*α*, whereas the acetylation of lysines 122 and 123 reduces its DNA binding affinity [69, 70]. p65 is the primary transcriptional activating component of NF-*κ*B and it appears that other NF-*κ*B subunits, such as p50, are expendable for function during liver injury and recovery [71, 72].

Lastly, the nuclear translocation and stability of the released NF-*κ*B appear to be regulated by the prolyl isomerase, Pin-1. Pin-1 is an isomerase that binds to the phosphorylated serine- or threonine-proline motif of its target proteins, and causes *cis*-*trans* isomerization about the peptidyl-prolyl bond [73]. Depending on the substrate, this can affect substrate's stability, cellular localization, activity level and its ability to interact with other proteins. Not surprisingly, Pin-1 has diverse roles in the cellular processes which are reflected in the diverse pool of substrates that include transcription factors, mitotic proteins and cytoskeletal proteins. NF-*κ*B p65 is one of the substrates of Pin-1, which binds to the phosphorylated threonine 254-proline motif of the p65 [74]. The binding of Pin-1 greatly stabilizes the NF-*κ*B complex by blocking the ubiquitin ligase-mediated degradation of p65. Furthermore, Pin-1 inhibits the binding of I*κ*B*α* to p65. This enhanced stability and inhibition of I*κ*B binding to p65 result in increased nuclear localization and prolonged activity of NF-*κ*B. The impact of Pin-1 in the activity of NF-*κ*B is seen in p65 mutants that have a threonine to alanine substitution at amino acid position 254. Such mutation prevents Pin-1 from binding to p65 and results in rapid degradation as well as failure of nuclear localization of NF-*κ*B [74]. The function of Pin-1 in liver I/R injury was recently confirmed in a mouse model in which it was shown that Pin-1 expression was requisite for adequate p65 stability [75]. In addition, this study showed that normothermic ischemia reduced Pin-1 expression in hepatocytes, but not Kupffer cells, suggesting a cell-specific regulation of NF-*κ*B activation during I/R injury by Pin-1. 

## 6. Hepatic Recruitment of Neutrophils

The propagation of inflammation in the liver after I/R by TNF*α* and, to a lesser degree, by IL-1 is accomplished through induction of the expression of adhesion molecules on vascular endothelial cells and stimulation of the production and release of CXC chemokines. Three classes of vascular cell adhesion molecules contribute to the adhesion and transmigration of neutrophils from the blood vessel lumen into the interstitial spaces. Selectins are glycoproteins expressed on endothelial cells (E- and P-selectins), platelets (P-selectin), and neutrophils (L-selectin) [76]. Selectins are responsible for leukocyte capture and transient adhesion to the vascular endothelium and all three family members have roles in leukocyte adhesion during hepatic I/R injury [77–80]. The increased neutrophil-endothelium interactions mediated by selectins facilitate the engagement of the other two classes of adhesion molecules, integrins and the immunoglobulin-like adhesion molecules. Integrins are expressed on the neutrophil surface (i.e., CD11b/CD18) and bind to immunoglobulin-like adhesion molecules that are expressed on the vascular endothelium (i.e., intercellular adhesion molecule-1, ICAM-1) [81]. These interactions mediate firm adhesion and transmigration and are essential for neutrophil recruitment into the liver after I/R [82, 83].

TNF*α* is clearly the primary stimulus for vascular cell adhesion molecule expression in the liver after I/R. TNF*α* is responsible for increasing hepatic vascular endothelial expression of P-selectin as well as ICAM-1 [43, 84]. P-selectin expression is not only important for the adhesion of neutrophils, but also for the adhesion of platelets to the hepatic endothelium [85]. Increased platelet accumulation within the hepatic microcirculation may enhance the subsequent adhesion of neutrophils, as adherent platelets are known to augment neutrophil adhesion at sites of inflammation [86]. More importantly, increased neutrophil accumulation has been attributed to sinusoidal endothelial cell injury, contributing to hepatic microvascular dysfunction [87]. Hepatic vascular expression of ICAM-1 is also increased by TNF*α*, and blockade of TNF*α* attenuates neutrophil accumulation and subsequent liver injury [38, 43].

In conjunction with vascular cell adhesion molecules, chemokines are an integral component of the process of neutrophil recruitment. Chemokines are a group of small (8–10 kD), basic, heparin-binding proteins that are secreted by leukocytes as well as various tissue cells [88, 89]. While mainly involved in leukocyte chemoattraction, chemokines have also been implicated in other cellular activities, including regulation of angiogenesis, fibrosis, proliferation, cytotoxicity, and apoptosis [90–93]. The nomenclature for chemokines is based on the configuration of a conserved amino-proximal cysteine-containing motif [94]. There are currently four branches of the chemokine family, CXC, CC, CX_3_C, and C (where X is any amino acid). CC and CXC are the two major branches, whereas CX_3_C and C each have only one representative, consisting of fractalkine (CX_3_CL1) and lymphotactin (XCL1), respectively [95]. The CC family is the largest, primarily involved in attracting mononuclear cells to sites of chronic inflammation, while members of the CXC family mediate the chemoattraction of neutrophils and monocytes to sites of acute inflammation [91]. CXC chemokines can be further classified by the presence or absence of a Glu-Leu-Arg (ELR) amino acid motif in the amino terminus of the peptide. The ELR motif confers receptor-binding specificity [96, 97].

CXC chemokines exert their effects through the CXC chemokine receptors (CXCR) 1–6 [95]. CXCR1 and CXCR2 bind specifically to CXC chemokines which contain the ELR motif  [90, 94]. In addition to their leukocyte-chemoattractant properties, ELR^+^ CXC chemokines have been shown to have important roles in angiogenesis and cellular proliferation [46, 92, 98]. CXCR1 and CXCR2 are expressed by neutrophils, monocytes, CD8+ T cells, epithelial cells, and endothelial cells, as well as in hepatocytes [99–101]. TNF*α* and IL-1 stimulate the production of a number of chemokines [94]. Chemokine production and/or presentation by endothelial cells activates neutrophils during their initial interactions with the vascular endothelium and promotes their subsequent firm adhesion. Hepatic production of chemokines forms a chemotactic gradient which serves to direct the recruitment of neutrophils into the injured liver. There appears to be selective differences in the expression of various CXC chemokines in the ischemic liver versus the nonischemic liver that causes the preferential recruitment of neutrophils into the ischemic liver [45]. CXC chemokines are also upregulated in remote organs, including lung, and play an important role in the development of remote organ injury after liver I/R [44, 46]. 

## 7. Neutrophil-Mediated Hepatocellular Injury

In liver, accumulation of activated neutrophils within the hepatic parenchyma causes hepatocyte damage through the release of oxidants and proteases. Effective killing of hepatocytes by neutrophils probably requires direct cell contact via CD11/CD18- and ICAM-1-dependent mechanisms [102, 103]. The primary neutrophil oxidant-generating pathway involves NADPH oxidase. Under normal conditions, this enzyme exists as inactive subunits located both on the cell membrane and in the cytoplasm. Cell activation causes translocation of cytosolic subunits to the cell membrane, resulting in assembly of a multimeric complex that exhibits oxidase activity. The active enzyme oxidizes NADPH and the released electron reduces molecular oxygen, forming O_2_^●^, superoxide anion. Further reduction of O_2_^●^ results in the generation of H_2_O_2_, which can be further reduced to the most active of all oxygen-centered radicals, the hydroxyl radical (HO^●^). HO^●^ can then be further reduced to H_2_O. Myeloperoxidase (MPO) released from neutrophil granules will, in the presence of a halide such as Cl^−^, enzymatically convert H_2_O_2_ to hypochlorous acid (HOCl), another potent oxidant. The generation of O_2_^●^, H_2_O_2_, HO^●^, and HOCl may directly damage hepatocytes [6, 104, 105] and/or deactivate endogenous antiproteases facilitating protease-mediated hepatocyte injury. 

In addition to the generation of oxidants, activated neutrophils release a number of mediators by granule exocytosis. The contents of neutrophil granules include large amounts of proteases (i.e., elastase, cathepsin G, heparanase, and collagenase) and hydrolytic enzymes that may be directly cytotoxic to hepatocytes [106, 107]. Serine proteases, such as elastase and cathepsin G may directly damage membrane components of hepatocytes, while metalloproteinases primarily degrade basement membrane and matrix components.

## 8. Modes of Hepatocyte Death after I/R

Apoptosis and necrosis are two distinct forms of cell death that differ morphologically. Necrotic cells are characterized by the loss of plasma membrane integrity and cellular architecture, vacuolization, and mitochondria swelling. Apoptotic cells, on the other hand, have as hallmarks chromatin condensation and nuclear fragmentation, cell shrinkage, and formation of apoptotic bodies. Although these two forms of cell death appear very different, they have some important similarities. First, mitochondrial dysfunction is a critical component of both forms of cell death. Specifically, opening of the nonselective permeability transition pores on the inner mitochondrial membrane leads to uncoupling of oxidative phosphorylation, membrane depolarization, and leaching of factors involved in cell death [108]. Second, both forms of cell death can be triggered by the same stimuli. In fact, intensity of a given stimulus and the intracellular ATP level appear to be important factors that determine whether a cell undergoes apoptosis or necrosis. An apoptotic stimulus can induce necrosis at higher intensity/concentration [109]. Alternatively, apoptotic stimuli can also cause necrosis if the intracellular ATP is depleted [110, 111]. There has been considerable debate about the primary mode of liver cell death after I/R. Some laboratories have reported substantial hepatocyte apoptosis [112], while others have shown that broad caspase inhibitors protect against I/R injury [113]. However, critical examination has shown that many of the parameters used to assess apoptosis in these studies also are positive in necrotic cells and that the final mode of death in the vast majority of hepatocytes after I/R is necrosis [114]. 

Because cells that would die by apoptosis undergo necrosis if intracellular ATP is depleted (a condition induced by prolonged ischemia), it is likely that necrotic cells seen after I/R may represent two populations: one population consisting of cells that incurred severe lethal damage and die by necrosis; a second population consisting of cells that initially were triggered to undergo apoptosis but, due to the lack of intracellular ATP, switched to necrotic cell death. Inhibition of apoptosis may provide protection against I/R injury by allowing the latter population of cells, that are injured but viable, a chance to survive and recover. This concept is supported by studies in which inhibition of apoptosis by overexpression of the antiapoptotic gene Bcl-2 reduced liver injury after I/R [115, 116].

Given the central role of TNF*α* in the injury response to hepatic I/R, it could serve as a primary stimulus for apoptosis. In fact, blocking TNF*α* production greatly reduces hepatic injury and apoptotic parameters after I/R, whereas the inhibition of Fas signaling had no effect on injury or evidence of apoptosis in this setting [117]. Similarly, TNF receptor-1 (TNFR-1)-knockout mice demonstrate less hepatic insult and apoptosis after I/R [117]. These findings suggest that TNF*α* may induce apoptotic signaling after I/R which may contribute to overall hepatocellular dysfunction and death. 

One of the best known functions of NF-*κ*B is its role in inhibiting apoptosis after TNF*α* exposure. The inflammatory and apoptotic response of TNF*α* is mediated through the signaling proteins that are recruited to TNFR-1 upon ligand binding. TNFR-1 associated death domain (TRADD) binds to the receptor and is involved in the transduction of both responses [118]. Binding of Fas-associated death domain protein (FADD) to TRADD leads to TNF-induced apoptosis that involves caspase 8 [119]. On the other hand, binding of TNFR-associated factor (TRAF)-2 and receptor-interacting protein (RIP) results in activation of NF-*κ*B and c-Jun NH_2_-terminal kinase (JNK). Although TNF*α* can elicit a potent apoptotic response, cells usually do not undergo apoptosis after exposure to TNF*α* because NF-*κ*B opposes this response. Failure to activate NF-*κ*B after TNF*α* stimulation results in apoptosis. For example, p65^−/−^ cells undergo apoptosis after TNF*α* stimulation. Cells that express a nonphosphorylatable form of I*κ*B fail to activate NF-*κ*B upon TNF*α* stimulation and also undergo apoptosis [120, 121]. Some of the most compelling evidence regarding the anti-apoptotic effects of NF-*κ*B came from the generation of p65 knockout mice, which die in utero due to massive hepatic apoptosis [122]. NF-*κ*B prevents apoptosis by transcribing a number of anti-apoptotic genes. For example, NF-*κ*B induces transcription of TRAF1 and 2 and inhibitory apoptotic protein (IAP)-1 and 2 which when expressed together block apoptosis by inhibiting caspase 8 activation [119]. When protein synthesis is blocked with cycloheximide, cells that are normally resistant to TNF*α* become susceptible, reflecting the fact that de novo synthesis of proteins induced by NF-*κ*B is required for cell survival [123]. Thus, NF-*κ*B may have a protective role after I/R by inhibiting apoptotic signaling and allowing injured but viable cells a chance to recover rather than succumb to secondary necrosis. 

## 9. Anti-Inflammatory Mediator Regulation of Liver Inflammation 

Like all homeostatic processes, regulatory mechanisms exist to help prevent a runaway inflammatory train that may otherwise lead to an overwhelming response. The degree of the insult often is directly proportional to the magnitude of the inflammatory response and with the evolution of advanced trauma stabilization and surgical procedures, patients are now surviving insults that in years past would have been lethal. While this is good for the patient initially, it sets the stage for an inflammatory response proportional to a lethal stimulus. In this setting, regulatory mechanisms are often overwhelmed and cannot effectively control the proinflammatory response. These regulatory mechanisms are often induced well after the initial insult and therefore represent a potential avenue for therapeutic intervention/supplementation. Our current knowledge of the endogenous mediators that regulate the hepatic inflammatory response is quite limited. For example, to date there have been few anti-inflammatory mediators identified that play substantial endogenous roles in control of the hepatic response to I/R. The cytokines IL-6, IL-10, and IL-13 have all been shown to be expressed during hepatic I/R injury [124–126]. However, only IL-6 and IL-13 appear to play important regulatory roles. IL-6 has been shown to limit hepatocellular injury and promote hepatocyte regeneration after I/R [126]. These effects of IL-6 were linked to its capacity to reduce the expression of TNF*α* and elaboration of c-reactive protein. IL-10, while expressed by the liver after I/R, does not appear to play a significant regulatory role [125]. Exogenous administration of IL-10, however, is highly protective and appears to suppress proinflammatory cytokine expression by inhibiting activation of the transcription factor, NF-*κ*B [127, 128]. The function of IL-13 is more complex. Exogenous administration of IL-13 prevents I/R injury by activating the transcription factor, STAT6, leading to blockade of transcriptional activation of the genes for TNF*α* and MIP-2 [129]. STAT6 was subsequently found to compete with NF-*κ*B for nuclear transcriptional coactivators and decreased NF-*κ*B transcription activation [130]. Studies of IL-13-knockout mice provide a different story to the role of this cytokine in the endogenous regulation of liver inflammation. IL-13 nullizygous mice display far more hepatocellular injury, but this occurred without significant alterations in NF-*κ*B activation, proinflammatory mediator expression, and coincided with a decrease in neutrophil accumulation [125]. These studies went on to show that endogenous IL-13 has multiple effects on the liver including a positive modulatory effect on expression of the adhesion molecule, vascular cell adhesion molecule-1 (VCAM-1). A decrease in hepatic VCAM-1 expression in IL-13-knockout mice was associated with a neutrophil transmigration defect leading to increased adhesion of neutrophils to the hepatic venular endothelium and increased hepatic endothelial cell injury. In addition, this study found IL-13 to directly protect cultured hepatocytes from oxidant-induced cytotoxicity [125]. 

The protease inhibitor, secretory leukocyte protease inhibitor (SLPI), has been shown to be a very potent endogenous regulator of the hepatic inflammatory response to I/R [131]. This small protein mediator was originally described as a secretory product of phagocytes that inhibited neutrophil elastase. It has subsequently been shown to be produced by a variety of cell types in various organs and has more complex functions, including inhibition of the transcription factor, NF-*κ*B [131–133]. Hepatic production of SLPI occurs prior to reperfusion, making somewhat unique amongst anti-inflammatory mediators, which are normally expressed after the initial surge of proinflammatory mediators. Endogenous SLPI appears to regulate liver inflammation by targeting the transcription factor, NF-*κ*B, and attenuating proinflammatory cytokine expression [131]. Additionally, its properties as a potent protease inhibitor may contribute to neutralization of destructive enzymes released by activated neutrophils [106, 133–136].

Nitric oxide (NO) has also been shown to be an important regulatory mediator in the liver. Its role in the injury response, however, has been somewhat controversial [137–147]. In the liver, NO is produced by both endothelial nitric oxide synthase (eNOS) as well as inducible nitric oxide synthase (iNOS), with the latter being expressed primarily in Kupffer cells and sinusoidal endothelial cells. Studies have shown that NO is produced by eNOS functions in a highly protective manner, with pharmacological inhibition strategies and eNOS-knockout mice showing greatly enhanced indices of hepatocellular injury, whereas production of NO from iNOS increases liver injury [148–151]. Currently, it is not clear whether eNOS-derived NO protects the liver by scavenging reactive oxygen species or by modulation of proinflammatory mediator expression.

## 10. Repair and Regeneration of Liver after I/R

Hepatocytes possess the unique ability to proliferate upon appropriate stimulation, normally maintaining themselves in a stage of quiescence, known as the G_0_ phase. The molecular basis of liver regeneration is composed of three different phases, including a priming phase, a proliferative phase, and a termination phase [152]. These phases have also been qualified as cytokine, growth factor, and metabolic pathways, respectively, as it pertains to the factors predominantly mediating a particular phase [153]. It is important to note that while it is conceptually easier to denote the sequence of events into “phases,” there is in fact a highly coordinated, synchronous schema of interactions between growth factors, cytokines, and other mediators that allow the process of liver regeneration to occur [154]. Cytokines are a key factor in stimulating quiescent hepatocytes from the G_0_ phase into the G_1_ phase. TNF*α* and IL-6, along with the transcription factors, STAT3 and NF-*κ*B, are required for the initiation of liver regeneration [153, 155]. Through activation of STAT3 and NF-*κ*B, target genes are transcribed which are important to hepatocyte proliferation. Growth factors, specifically hepatocyte growth factor (HGF) and epidermal growth factor (EGF), then drive the cell from G_1_ into the S phase of DNA replication [153]. Arguably one of the most important mediators of liver regeneration is HGF—a 100 kDa protein that was originally identified in 1984 [156, 157]. A potent mitogen for hepatocyte growth, HGF, is locally released and upregulated during the initiation of the regenerative process, cleaved from its inactive single-chain form into its active two-chain form by uPA [152].

Phospholipase C*γ*1 (PLC*γ*1), phospholipase C*β*1 (PLC*β*1), phospholipase D1 (PLD1), and phosphoinositide-3-kinase (PI3K) have been implicated in the mechanisms of hepatocyte proliferation immediately after HGF or EGF binding [158–161]. PLC*γ*1 and PLC*β*1 appear to play different roles in the regenerating liver, with PLC*γ*1 having more influence on the G_2_/M phase transition, and PLC*β*1 seeming to trigger DNA replication [158]. PLD1 may play a role in the activation of c-Jun/c-Fos transcription factors, further contributing to DNA synthesis [162]. The HGF receptor, a c-met oncogene, has been shown to function through tyrosine kinase activity. However, Adachi et al. [163], showed that pertussis toxin-sensitive G proteins were also involved in mitogen activated protein kinase (MAPK) activation and arachidonic acid release, specifically demonstrating that PLD activation was diminished to baseline levels in the presence of G*α*_i_ receptor complex inhibition. More recently, signaling through PI3K has been shown to be critical for the induction of cyclin D and DNA replication following HGF binding [161]. Further downstream, MAPK-dependent production of arachidonic acid (AA) through PLA_2_ results in production of prostaglandins, further stimulating DNA synthesis [160]. Prostaglandins, most significantly PGE_2_ and PGF_2_, are known to promote growth in hepatocytes [164]. Conversely, during conditions in which hepatocytes may be stressed, activation of PLA_2_ and increased release of arachidonic acid may have a deleterious effect on hepatocytes [165]. In the setting of hypoxic injury to hepatocytes, diminished ATP production leads to acidosis, therefore preventing activation of PLA_2_ until the return to physiologic pH during reperfusion, resulting in AA release and increased cell death [165, 166]. In vivo studies have revealed that COX-2-dependent conversion of arachidonic acid to prostaglandins is crucial to the induction of protective mechanisms within the liver, and that COX-2 inhibition contributed to greater hepatotoxicity in the setting of carbon tetrachloride (CCl_4_) injury, perhaps indicating that the level of COX-2 following hepatic injury is important to recovery [167].

## 11. Contribution of CXC Chemokines to Liver Repair and Regeneration

As described above, CXC chemokines are known to be important mediators of the inflammatory cascade following hepatic injury and also appear to have a dichotomous role in hepatocytes that may be related to their level of expression [168]. For example, induction of CXC chemokines at relatively low levels is associated with liver repair and regeneration, whereas high expression levels have been associated with hepatotoxicity [101, 169–171]. The impact of CXC chemokines on the regenerative capacity of the liver was first examined using an in vivo model of partial hepatectomy [169]. Partial hepatectomy represents a clinically relevant model of hepatic resection, a procedure often performed due to trauma or malignancy. ELR^+^ CXC chemokines are upregulated after partial hepatectomy, and blockade of chemokines or CXCR2 results in diminished liver regeneration [169]. Subsequent in vitro experiments demonstrated that hepatocytes treated with ELR^+^ CXC chemokines proliferated to a degree similar to that induced by HGF. These studies [101, 169–171] provided evidence that ELR^+^ CXC chemokines were important hepatocyte proliferative factors that functioned in vivo to promote liver regeneration after hepatectomy. However, as previously mentioned, the remnant liver after resection or hepatectomy, without Pringle maneuver, is comprised of unstressed hepatocytes. The role of CXC chemokines may be distinctly different in a setting in which hepatocytes are under significant stress, such as I/R.

Liver recovery and repair after I/R injury in this model begins approximately 48 hours after reperfusion and is associated with increased expression of stathmin and marked hepatocyte proliferation [172]. Liver repair and regeneration typically return the liver to its normal, homeostatic state within 5–7 days after reperfusion, depending on the severity of the injury. It is during this reparative/regenerative phase that it appears that the function of CXC chemokines switches from a proinflammatory role to direct impingement on hepatocyte proliferation or death. Knockout of CXCR2, the primary receptor for ELR^+^ CXC chemokines in rodents, resulted in accelerated liver recovery associated with increased activation of NF-*κ*B and STAT3 transcription factors resulting in increased hepatocyte proliferation [170]. Antibody blockade of CXCR2 after induction of I/R injury had the same effect [170]. These studies suggest that during the reparative/regenerative phase of I/R injury, ELR^+^ CXC chemokines have harmful effects which delay liver recovery. 

These apparent harmful effects of ELR^+^ CXC chemokines are likely a result of specific signaling via CXCR2 in hepatocytes. While the presence and involvement of CXCR2 in murine models of hepatocyte injury and regeneration have been well characterized [169, 170], murine CXCR1 has only been recently identified [173, 174]. Previous work has demonstrated that CXCR2 is constitutively expressed in hepatocytes [101] and may be upregulated in the presence of certain cytokines [175]. While CXCR2 and its ligands appear to play a key role in hepatocyte proliferation following partial hepatectomy, and hepatocyte toxicity following I/R injury or acetaminophen toxicity, the role of CXCR1 is less clear. CXCR1 is not constitutively expressed in the liver [173]. This finding was recently confirmed, but CXCR1 were found to be induced in hepatocytes after I/R [100]. Blockade and knockout of CXCR1 was found to result in delayed liver repair after I/R, although there were no observed changes in hepatocyte proliferation in vivo [100]. While the effects of CXCR1 blockade or knockout on liver repair were not as striking as those observed with CXCR2, the findings suggest that CXCR1 has a divergent function in hepatocytes, compared to CXCR2. 

While the stress level of the hepatocyte may alter its response to CXC chemokines, so may the concentration of available ligand. Following 70% partial hepatectomy, expression of ELR^+^ CXC chemokines increases approximately 5-fold [169], whereas after I/R it increases hundreds- to thousandsfold [170]. In vitro, stimulation of primary hepatocytes with CXC chemokines has hepatoprotective effects at low concentrations and progressively cytotoxic effects at increasingly greater concentrations, effects which are specific to CXCR2 [100, 170]. Adenoviral-mediated liver overexpression (>100-fold) of the CXC chemokine, keratinocyte-derived chemokine, has been shown to result in massive hepatocellular necrosis within 48 hours [71]. Collectively, these studies suggest that moderate increases in CXCR2 ligands, occuring after partial hepatectomy may promote liver regeneration, whereas much larger increases in expression of CXCR2 ligands, occuring after I/R injury, may be hepatotoxic and/or oppose hepatocyte proliferation and regeneration.

## 12. Conclusions

Hepatic I/R injury is a primary complication of liver resection and transplantation and is also a consideration during vascular and trauma surgery. The impact of this injury on patient morbidity and mortality is significant. Experimental studies have identified the primary mechanisms of this injury response, which begins as an oxidative stress and culminates in a robust inflammatory response leading to neutrophil-mediated injury to hepatocytes. This entire process is regulated largely by inflammatory cytokines and is regulated by endogenous expression of anti-inflammatory mediators that serve to resolve the response. An equally complex process for tissue repair and regeneration of lost functional mass includes participation of several proinflammatory mediators, such as CXC chemokines, which also serve as secondary mitogens for hepatocytes. Collectively, these experimental findings have helped identify many new therapeutic targets that can help reduce the incidence of and mitigate the impact of I/R injury to the liver to improve patient care.
